# A Common Origin for Immunity and Digestion

**DOI:** 10.3389/fimmu.2015.00072

**Published:** 2015-02-19

**Authors:** Nichole A. Broderick

**Affiliations:** ^1^Department of Molecular, Cellular, and Developmental Biology, Yale University, New Haven, CT, USA

**Keywords:** innate immunity, host-microbe interactions, gut microbiome, evolution, metabolism, digestion physiology

## Abstract

Historically, the digestive and immune systems were viewed and studied as separate entities. However, there are remarkable similarities and shared functions in both nutrient acquisition and host defense. Here, I propose a common origin for both systems. This association provides a new prism for viewing the emergence and evolution of host defense mechanisms.

The immune system appeared early in the evolution of metazoa and is thought to have originated to protect the greater investment of multi-cellularity (1, 2). Currently, two views have been suggested for the origin of the immune system. One view is that the immune system emerged to protect against invasive microbes (3, 4). Recently, an alternative hypothesis proposed that the immune system emerged to manage the microbiota (5–7). In this essay, I suggest a third selection pressure, not exclusionary of the other views that focus on function. I propose a common origin for the digestive and immune systems that traces their ancestry to the quest for more efficient energy acquisition.

## In the Beginning, Digestion and Immunity were One …

Over a century ago, Metchnikoff proposed that phagocytic immune cells evolved first as nutritive cells (8). He noted that, across phylogeny, the universal function of digestion was maintained in the process of phagocytosis, irrespective of whether it occurred in the case of food acquisition (intracellular digestion) or in an immune role. Moreover, for single-celled organisms like amoebae, the process of infection and food acquisition are indistinguishable in the initial stages, with the two being separable only by outcome. The evolution of multi-cellularity permitted specialization, including cells devoted to the acquisition of nutrients and defense. In the event of infection, phagocytic cells ingest, destroy, and digest microbes resulting in an outcome that is indistinguishable from single-celled predation. As proposed by Metchnikoff, these cells retain the primordial mechanism of nutrient acquisition, providing continuity between nutritional and defensive roles.

While Metchnikoff saw the connection between digestion and defense, the immune system is composed of many diverse processes beyond the cellular response of phagocytosis and indeed animals most commonly interact with the microbes in their environment through contact with epithelial surfaces. Analysis of such immune and digestive components across animals reveals several remarkable parallels (Table 1), with many enzymes involved in immune responses also having roles in digestion. Given the gut was a major early step in the evolution of metazoa, this tissue is a logical starting point for an analysis of common function.

**Table 1:** **Examples of dual-use action in digestion and immunity**.

Component	Type	Digestion/metabolism	Immunity
Enzymes	Proteases	Protein break down	IgA cleavage, toll signal processing
	Lysozymes	Cell wall break down	Microbial lysis
	Chitinases	Chitin digestion	Microbial lysis, augment adaptive responses, wound healing
	Phenoloxidases	Lignin degradation (fungi, invertebrates)	Melanin synthesis
	β1,3-glucanases	Sugar break down	Pattern recognition receptor
	Amidases	Cell wall break down	Microbial lysis
	Antimicrobial peptides	Cellular break down	Microbial lysis
Receptors/signaling	TIR domain proteins	Foraging	Toxin sequestration
		Response to nutrients	Immune effectors, pathogen avoidance
		Starvation resistance	Pattern recognition receptor
Cellular processes	Phagocytosis autophagy	Food acquisition	Microbial clearance
		Intracellular digestion of food	Microbial clearance

## The Gut – Conservation and Divergence of Digestive and Immune Functions

The gut is ancient in the animal lineage and arose shortly after the emergence of multi-cellularity. The gut is thought to have begun with the formation of a true epithelium, which allowed extracellular digestion, followed by invagination of the epithelium to provide an enclosed space to facilitate the digestive process. The gut later progressed from a one-way digestive tube to the highly organized and specialized organ that is found today in most animals [for more details on the evolution of the gut, see Ref. (9, 10) and references therein]. Multiple evolutionary advances of metazoa are attributed to the development and adaptation of the gut (9), as it permitted extracellular digestion and the capacity to digest larger volumes without losing nutrients to diffusion (11). Consequently, the emergence of the gut is thought to have increased energy availability, which in turn may have driven the development of other organ systems.

The gut evolved in a sea of microbes, which posed new challenges and provided new opportunities for nutrition (7). In its most primitive stages, the gut would have provided a new niche that allowed or even invited colonization by microbes. Hosts had to contend with these microbes, either through indifference, forming beneficial associations, eating them, or controlling them to reduce microbial-mediated damage and/or competition for nutrients. While both invertebrates and vertebrates possess innate immune functions, only the latter have adaptive immunity. For this reason, it is thought that the evolution of the immune system paralleled the evolution of the gut, with immunological complexity emerging to protect an increasingly sophisticated digestive tract. In contrast, this essay proposes the alternative view that innate immune defense and digestion were indistinguishable in the primitive gut.

This hypothesis has a number of implications for our understanding of the evolution of the immune system. What today are presumed to be disparate primordial functions may not be so. For example, one immediate implication is that intersections between immunity and metabolism may be more intricately linked than previously appreciated (12, 13). An immediate parallel is that both pathogen clearance and digestion involve microbial destruction. Hence, enzymes produced for digestion have dual-use function in protection and subsequent evolution of the genes could promote specialization and divergence. For example, β1,3-glucanases are digestive enzymes, but one subfamily lost catalytic activity and is a pattern recognition receptor. Similarly, toll-interleukin-1 receptor (TIR) domain proteins in amoebae and an ortholog in *C. elegans* have dual roles in nutritional foraging and defense (14, 15). These dual roles suggest contexts when these functions cannot be distinguished. This may be especially true amongst animals where bacterivory, or the feeding on bacteria as an energy source, provides a major source of nutrition. The role of bacterivory in the origin and evolution of animals is important due to its potential in providing for the acquisition of novel functions through lateral gene transfer (16, 17). In this regard, lateral transfer of genes with putative defensive function could have been maintained due to their role as digestive enzymes. For example, two recent reports of genes transferred from bacteria to eukaryotes that were associated with antibacterial activity (one a lysozyme, the other an amidase) could easily function in the digestion of bacteria for nutrient acquisition (18, 19). In this regard, lysozymes have historically been categorized into groups as either having defensive or digestive functions, yet they are essentially identical. By and large, the digestive and immune functions of these enzymes has been assigned based on the tissues in which they are expressed, gut or stomach versus immune cells, respectively. However, recent studies have argued that they were preserved through common descent with true homology (20, 21).

This hypothesis also suggests alternative views to how the microbiota and the defensive function of the immune system evolved. Considering the emergence of the microbiota, one might envision how microbes that were not digested could be maintained in the gut. In this regard, microbes that contributed to host nutrition by producing a byproduct or transforming compounds could reduce host requirements for microbes as direct food sources, thus permitting their retention. In addition, microbes that are able to resist the digestive processes would have been able to persist in the gut and form potential associations with the host. Alternatively, hosts that evolved gut attributes (physical/physiological) or the ability to selectively digest microbes would be able to maintain a microbiota, which would have been selected for if it provided an advantage. McFall-Ngai (22) has proposed that the evolution of the adaptive immune system may have permitted greater flexibility in the diversity of microbes associated with the gut. Along these lines, the reduced reliance of microbes as direct food sources may have permitted greater diversity of the microbiota and further specialization of epithelial immune responses. In addition, the microbiota is a potential food reserve. Axenic mice are more susceptible to starvation, and starvation of many animals reduces microbiota density, suggesting utilization as food (23–25) Moreover, such phenomenon as termite trophallaxis and digestion of microbiota by nitrogen-deprived herbivores supports the notion that the microbiota can provide a nutritional reserve (26). It is noteworthy that there are 20% more calories in a gram of microbes than a gram of carbohydrate (27). However, the same microbiota that can serve as food also poses a potential danger to the host as a source of infectious disease. Consequently, these interactions between host and microbiota illustrate the continuity between digestion and immunity.

## Conclusion

This hypothesis proposes a common origin for two fundamental physiological systems that are currently viewed as separate and disparate. While the interplay between metabolic and immune pathways, including genes that function in both systems (28) [i.e., *foxo* (29), *MyD88* (30, 31), *TGF-*β (32), *mef2* (33), *atf3* (34), …] is an area of intense study, these similarities are generally viewed as convergent. In contrast, this hypothesis posits a common origin for these functions, thus providing an explanation for the maintenance of dual functions. I note similar associations in other systems, such as *Toll* having roles in both immunity and development. Underlying these associations is the fact that the ancient function of proteins might be conserved, but can also be co-opted for new roles.

The hypothesis has some practical applications for the interpretation of experiments involving mutants of either metabolic or immune pathways. For example, phenotypes attributed to immune deficiencies, which are often associated with higher microbial burden, could also represent a digestive or metabolic deficiency. An approach to test the hypothesis may be to delete genes that are putatively associated with an immune or digestive function and assess the resulting phenotypes of the other system. By considering a common origin for immunity and digestion, it is possible to integrate metabolic, physiological, and immune information and interpret those data in the context of a unified view.

## Conflict of Interest Statement

The author declares that the research was conducted in the absence of any commercial or financial relationships that could be construed as a potential conflict of interest.
